# Characteristic brain function and network activity patterns in adolescent first-episode depression: a resting-state functional magnetic resonance imaging study

**DOI:** 10.3389/fnimg.2025.1677410

**Published:** 2025-11-28

**Authors:** Liulu Zhang, Pingping Jie, Jie Zhao, Yuting Fu, Yong Liu, Bo Xiang, Jun Lv, Weidan Luo

**Affiliations:** 1 Department of Magnetic Resonance, The Affiliated Traditional Chinese Medicine Hospital, Southwest Medical University, Luzhou, Sichuan, China; 2 Department of Psychiatry, Fundamental and Clinical Research on Mental Disorders Key Laboratory of Luzhou, Medical Laboratory Center, Affiliated Hospital of Southwest Medical University, Luzhou, Sichuan, China; 3 General Internal Medicine, The Affiliated Traditional Chinese Medicine Hospital, Southwest Medical University, Luzhou, Sichuan, China; 4 Department of Vascular Surgery, The Affiliated Hospital of Southwest Medical University, Southwest Medical University, Luzhou, Sichuan, China

**Keywords:** adolescent, first-episode depression, resting-state fMRI, brain function, network activity patterns

## Abstract

**Background:**

The characteristic brain function and network activity patterns in adolescents with first-episode depression (FED) remain systematically underexplored. This study aims to investigate abnormalities in cerebral function and networks in adolescent FED patients through analyses of the amplitude of low-frequency fluctuations (ALFF), fractional amplitude of low-frequency fluctuations (fALFF), and independent component analysis (ICA).

**Materials and methods:**

A cohort of 36 adolescents with first-episode depression (patient group, PT) and 34 healthy controls (HC group) were enrolled. Depressive symptoms were assessed using the Hamilton Depression Rating Scale (HAMD) and Children’s Depression Inventory (CDI). All participants underwent resting-state functional magnetic resonance imaging (rs-fMRI). Neuronal activity and functional network alterations were analyzed via ALFF, fALFF, and ICA methodologies.

**Results:**

Compared to the HC group, the PT group exhibited increased ALFF values in the left fusiform gyrus (Fusiform_L), left middle temporal gyrus (Temporal_Mid_L), right middle occipital gyrus (Occipital_Mid_R), right middle temporal gyrus (Temporal_Mid_R), right calcarine cortex (Calcarine_R), right angular gyrus (Angular_R), and left calcarine cortex (Calcarine_L). Elevated fALFF values were observed in the right calcarine cortex (Calcarine_R) and left superior temporal gyrus (Temporal_Sup_L), while decreased fALFF values were detected in the left superior temporal pole (Temporal_Pole_Sup_L), right medial superior frontal gyrus (Frontal_Sup_Medial_R), left superior frontal gyrus (Frontal_Sup_L), and left precuneus (Precuneus_L). Connectivity differences within the visual network (VIN) were identified between groups, with a peak difference in the right inferior temporal gyrus (Temporal_Inf_R), where the PT group demonstrated hyperconnectivity.

**Conclusion:**

In summary, neurofunctional abnormalities in adolescent FED patients involve the temporal lobe emotion-processing network, prefrontal executive control system, and default mode network (DMN). Aberrant low-frequency activity in the temporal pole and superior frontal gyrus may exacerbate emotion dysregulation, whereas hyperactivation of the precuneus and visual cortex could potentiate negative self-referential processing. Notably, the right middle occipital gyrus may represent a distinctive biomarker of adolescent depression. These findings provide novel insights into the early neural mechanisms underlying adolescent depression and suggest that non-invasive neuromodulation techniques targeting specific brain regions (e.g., transcranial magnetic stimulation, TMS) hold therapeutic potential.

## 1 Introduction

Depression is a mental disorder characterized by persistent sadness and/or guilt persisting for at least 2 weeks, diminished interest in activities, or accompanied by associated impairments in daily and social functioning (Gore et al., 2011; GBD 2021 Diseases and Injuries Collaborators, 2024). Adolescence constitutes a critical period for neurodevelopment and psychosocial adaptation, while also representing the peak period of depression onset. Epidemiological data indicate that approximately 11–20% of adolescents worldwide are affected by depressive disorders, with first-episode depression (FED) patients demonstrating elevated risks of chronicity and a propensity for cognitive impairment (Chen et al., 2025). While functional magnetic resonance imaging (fMRI) studies based on adult samples have begun to elucidate functional connectivity abnormalities in hippocampal circuitry among depression patients, the distinctive neuroplasticity features of adolescent populations may confer marked heterogeneity in their pathological mechanisms (Li et al., 2025).

Resting-state fMRI (rs-fMRI) studies have started to delineate the neurobiological underpinnings of adolescent MDD. Metrics of local brain activity, such as the amplitude of low-frequency fluctuations (ALFF) and fractional ALFF (fALFF), have revealed aberrant neural activity in key regions. For instance, studies have consistently reported increased ALFF/fALFF in the default mode network (DMN) components, such as the dorsal-medial prefrontal cortex and middle cingulate cortex, which is thought to underlie the negative self-referential thinking and rumination characteristic of depression (Guo and Wei, 2025). Conversely, decreased activity in the central executive network (CEN), including the dorsolateral prefrontal cortex, is frequently observed and linked to impaired cognitive control and emotion regulation (Wang et al., 2024). Alterations are also noted in limbic and paralimbic areas; for example, hyperactivity in the amygdala and insula—core nodes of the salience network (SN)—may reflect heightened sensitivity to negative emotional stimuli (Macdonald et al., 2025). Furthermore, functional connectivity analyses have demonstrated dysregulated interactions between these large-scale networks, particularly DMN hyperconnectivity and compromised CEN-DMN anticorrelation, which is hypothesized to disrupt the balance between internal thought and external attention (Kaiser et al., 2015). However, findings remain heterogeneous, and many prior studies have focused on a single analytical perspective, either local activity or network connectivity, in isolation.

Recent multimodal neuroimaging evidence reveals developmental-specific manifestations in adolescent first-episode depression (MDD), characterized by hyperactivation of the default mode network (DMN) and functional decoupling of the central executive network (CEN). This disruption of dynamic inter-network equilibrium strongly correlates with emotional dysregulation and negative cognitive bias (Liang et al., 2025). A diffusion tensor imaging (DTI) study integrating white matter microstructural alterations demonstrated significant segmental differences in major white matter tracts among MDD patients. Tract-based spatial statistics (TBSS) analysis identified reduced fractional anisotropy (FA) in the cingulum bundle, forceps minor, inferior fronto-occipital fasciculus, inferior longitudinal fasciculus, superior longitudinal fasciculus (SLF), and uncinate fasciculus, delineating microstructural pathology in adolescent MDD (Zhang et al., 2025).

Functional magnetic resonance imaging (fMRI) utilizing blood oxygen level-dependent (BOLD) sequences has become instrumental in psychiatric research. A study by Liu et al. investigating brain regional alterations in depressed female adolescents with suicidal ideation revealed decreased fractional amplitude of low-frequency fluctuation (fALFF) and regional homogeneity (ReHo) in the left middle frontal gyrus compared to healthy controls. The suicidal depression group exhibited enhanced functional connectivity (FC) between the right precentral gyrus and left middle frontal gyrus/left insula, as well as between the right insula and anterior/mid-cingulate cortex (Liu et al., 2022).

However, a comprehensive understanding of the pathophysiological mechanisms of adolescent FED requires an integrative approach that simultaneously characterizes both local neural activity and system-level network dynamics. Most existing studies have employed a single rs-fMRI metric, thereby providing a fragmented view of the brain’s functional alterations. This study employs a multi-parametric resting-state fMRI approach to systematically investigate aberrant functional architecture and network dynamics in adolescent MDD, establishing dynamic correlations with clinical symptom dimensions. These findings aim to advance early identification systems and targeted interventions grounded in neuroimaging biomarkers.

## 2 Materials and methods

### 2.1 Study participants

The study included 36 medication-naïve first-episode depression patients (PT group) and 34 demographically matched healthy controls (HC group), recruited from outpatient clinics of Southwest Medical University Affiliated Hospital and Traditional Chinese Medicine Hospital (2022–2023). Inclusion criteria for PT group: (1) met Diagnostic and Statistical Manual of Mental Disorders, Fifth Edition (DSM-V) diagnostic criteria for depression; (2) Hamilton Depression Scale (HAMD) 17-item scores of ≥17; CDI > 8; (3) subjects had not received antidepressant medication or experienced clearance of at least five half-lives of previously prescribed medication; and (4) were between 12 and 18 years of age, adolescent age; (5) right-handed. Exclusion criteria for all participants: (1) Comorbid neuropsychiatric disorders; (2) Substance abuse (caffeine/nicotine/alcohol) history; (3) MRI contraindications; (4) Structural brain abnormalities on T1/T2-FLAIR MRI; (5) Pregnancy.

Healthy control (HC) participants were relatively matched for gender, age, and education, and inclusion criteria were: (1) Minor, age 12–18 years old; (2) right-handed; (3) good sleep quality, no history of staying up all night within 1 week; (4) no psychiatric or neurological diseases; (5) no contraindications to MRI examination and no abnormal signals confirmed by cranial routine T1 or T2 fluid-attenuated inversion recovery (FLAIR) MRI; (6) HAMD score ≤ 7, CDI ≤ 8; (7) no history of staying up late and drinking large amounts of alcohol, coffee, and other stimulating foods within 2 days prior to MRI; and There was no history of staying up late, drinking a lot of alcohol, drinking strong tea, coffee and other stimulating foods within 2 days. All subjects gave written informed consent in accordance with the Declaration of Helsinki. The study was approved by the Ethics Committee of the Affiliated Hospital of Traditional Chinese Medicine of Southwest Medical University (KY2022069).

### 2.2 MRI sequences

Siemens Skyra 3.0 T MRI (Siemens Magnetom Verio; Siemens Medical Systems, Erlangen, Germany) with a 16-channel combined head and neck coil was used for data acquisition, and routine T1W and T2W were performed before resting-state data acquisition, T2-FLAIR sequences were performed to exclude cerebral hemorrhage, infarction, and tumor. During the resting state scanning, the subjects were instructed to lie still, breathe calmly with eyes closed, and try not to perform any thinking movement, fix the head with foam pads to reduce head movement, and wear earplugs to reduce the noise, and the scanning was started after the subjects were familiarized with the environment. Functional imaging of resting cerebral oxygen-dependent levels was acquired using a Gradient Recalled Echo (GRE) sequence with the following parameters: transition time (TR) = 2,000 ms, echo time (TE) = 30 ms, flip angle 90°, thickness/gap = 3.5/0 mm, field of view (FOV) = 1.5/0 mm, and field of view (FOV) = 1.5/0 mm. field of view(FOV) = 224 mm × 224 mm, number of layers 32, acquisition time point 200, and acquisition time 6min46s. In addition, anatomical T1-weighted whole brain magnetization-prepared rapid gradient-echo (MPRAGE) images acquisition parameters: repetition time (TR) = 2,530 ms, echo time (TE) = 2.98 ms, flip angle 90°, thickness/gap = 1.0/0 mm, field of view (FOV) = 256 mm × 256 m, number of sagittal slice layers 176.

### 2.3 Data preprocessing

Data preprocessing was performed using the RESTplus V1.2 toolbox^1^ based on the MATLAB R2023b platform, following the pipeline described by Zhang et al. (2025). The steps were as follows: (1) the first 10 time points were removed to allow for magnetic field stabilization and participants’ adaptation to the environment, and the subsequent 190 time points were retained for analysis. (2) Slice timing correction was applied to account for acquisition time differences between slices. (3) Head motion correction was performed. Participants with maximum translation > 2.5 mm or rotation > 2.5° in any direction were excluded from further analysis. (4) Spatial normalization was conducted by co-registering the BOLD images to each participant’s corresponding T1-weighted structural image, which was then normalized to the Montreal Neurological Institute (MNI) space using the DARTEL algorithm. The functional images were subsequently resampled to a voxel size of 3 × 3 × 3 mm^3^. (5) Spatial smoothing was applied with a 6 mm full-width at half-maximum (FWHM) Gaussian kernel. (6) Linear detrending was performed to remove slow drifts in the signal. (7) Nuisance covariate regression was carried out to remove the potential effects of the mean signals from white matter and cerebrospinal fluid, as well as the 24 Friston head motion parameters. This approach helps to mitigate the influence of residual micromovements that persist after the exclusion of high-motion subjects. (8) Finally, a band-pass filter (0.01–0.08 Hz) was applied to the time series to reduce low-frequency drift and high-frequency physiological noise. Global signal regression was not performed.

### 2.4 Calculation of ALFF metrics

ALFF was calculated for each voxel in the 0.01–0.08 Hz frequency band and normalized by the z-score for the whole brain, i.e., each voxel value was subtracted from the whole-brain mean divided by the whole-brain standard deviation to obtain the zALFF.

### 2.5 ICA analysis

Group-level spatial independent component analysis (ICA) was performed on the preprocessed fMRI data using the GIFT toolbox.^2^ The number of independent components (ICs) was automatically estimated to be 30 using the Minimum Description Length (MDL) criterion, which provides a data-driven estimate of the optimal dimensionality for decomposing the resting-state data. Principal component analysis was used for data reduction, decomposing the subject-specific data into 45 principal components, which were then further decomposed into 30 group-level ICs using the infomax algorithm. The stability of the ICA decomposition was verified using the ICASSO toolbox. Subsequently, group-level ICs were back-reconstructed to generate subject-specific spatial maps and time courses using the GICA method. From the resulting 30 components, functional networks (FNs) of interest were identified and selected through a two-step procedure: visual inspection of their spatial patterns to ensure they represented recognizable, large-scale functional networks rather than noise (e.g., artifacts from head motion, ventricles, or white matter). Based on these criteria, 11 components that robustly corresponded to 8 well-characterized resting-state networks (e.g., Default Mode Network, Visual Network) were selected for subsequent group-level statistical analysis.

### 2.6 Statistical analysis

Demographic and clinical data were analyzed using SPSS 26.0. An independent-samples t-test was used for age comparisons. The chi-square test was used for gender comparisons. Given the non-normal distribution of the clinical scale scores, the Mann–Whitney U test was used to compare HAMD and CDI scores between groups. Voxel-wise group analyses of the neuroimaging data were performed using two-sample t-tests in SPM12. Age, gender, and mean framewise displacement were included as covariates of no interest in all models to control for potential confounding effects. The following multiple comparison correction strategies were applied: For the ALFF and fALFF analyses, statistical significance was assessed using voxel-wise False Discovery Rate (FDR) correction with a significance level of *p* < 0.05. For the ICA-based functional connectivity analysis, the statistical threshold was set at an uncorrected voxel-level height threshold of *p* < 0.001, with cluster-level inference using Family-Wise Error (FWE) correction at *p* < 0.05. Regions surviving these corrections were defined as statistically significant. The peak points (Peak points) in the statistically significant regions of the differences were analyzed by the Montreal Coordinate System (Montreal Neurological Montreal Neurological Institute; MNI) coordinates of the peak point (Peak point) in the statistically significant region were localized and named as statistically significant brain regions.

## 3 Results

### 3.1 Results of general information analysis

In the depressed PT group and the healthy HC group, no significant differences in age and gender were observed between the two groups (*p* > 0.05, Table 1), and there were significant differences in HAMD and CDI scale scores between the two groups (*p* < 0.001, Table 1).

**Table 1:** Results of the analysis of general information.

	PT group	HC group	*p* value
Age, year	15.50 ± 1.76	16.09 ± 1.45	>0.05
Gender, *n* (M/F)	5/21, 36	5/19, 34	>0.05
HAMD	29.00 (38.00, 23.25)	0.00 (0.25, 0.00)	<0.001
CDI	34.00 (38.00, 29.00)	7.00 (8.25, 3.00)	<0.001

### 3.2 Differences in ALFF between the two groups

**Figure 1:**
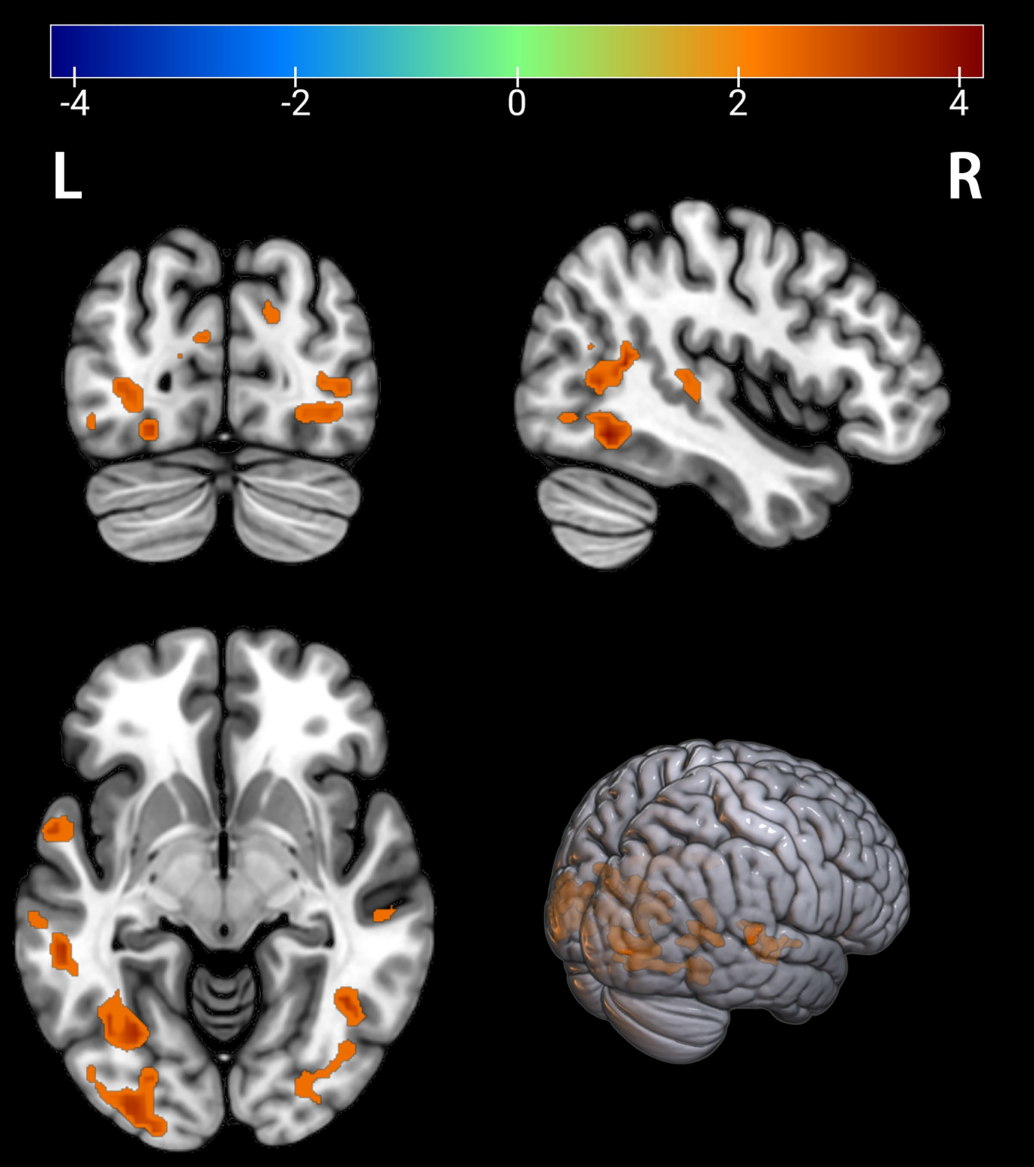
Brain regions with significantly increased ALFF in the PT group compared to the HC group. The results are displayed on a 3D brain template. Statistical significance was determined by a two-sample t-test, with voxel-wise FDR correction at *p* < 0.05. The color bar represents the T-value. Warm colors (red) indicate a significant increase in ALFF in the PT group. Significant clusters include the left fusiform gyrus (Fusiform_L), left middle temporal gyrus (Temporal_Mid_L), right middle occipital gyrus (Occipital_Mid_R), right middle temporal gyrus (Temporal_Mid_R), right calcarine cortex (Calcarine_R), right angular gyrus (Angular_R), and left calcarine cortex (Calcarine_L). R, right; L, left.

**Figure 2:**
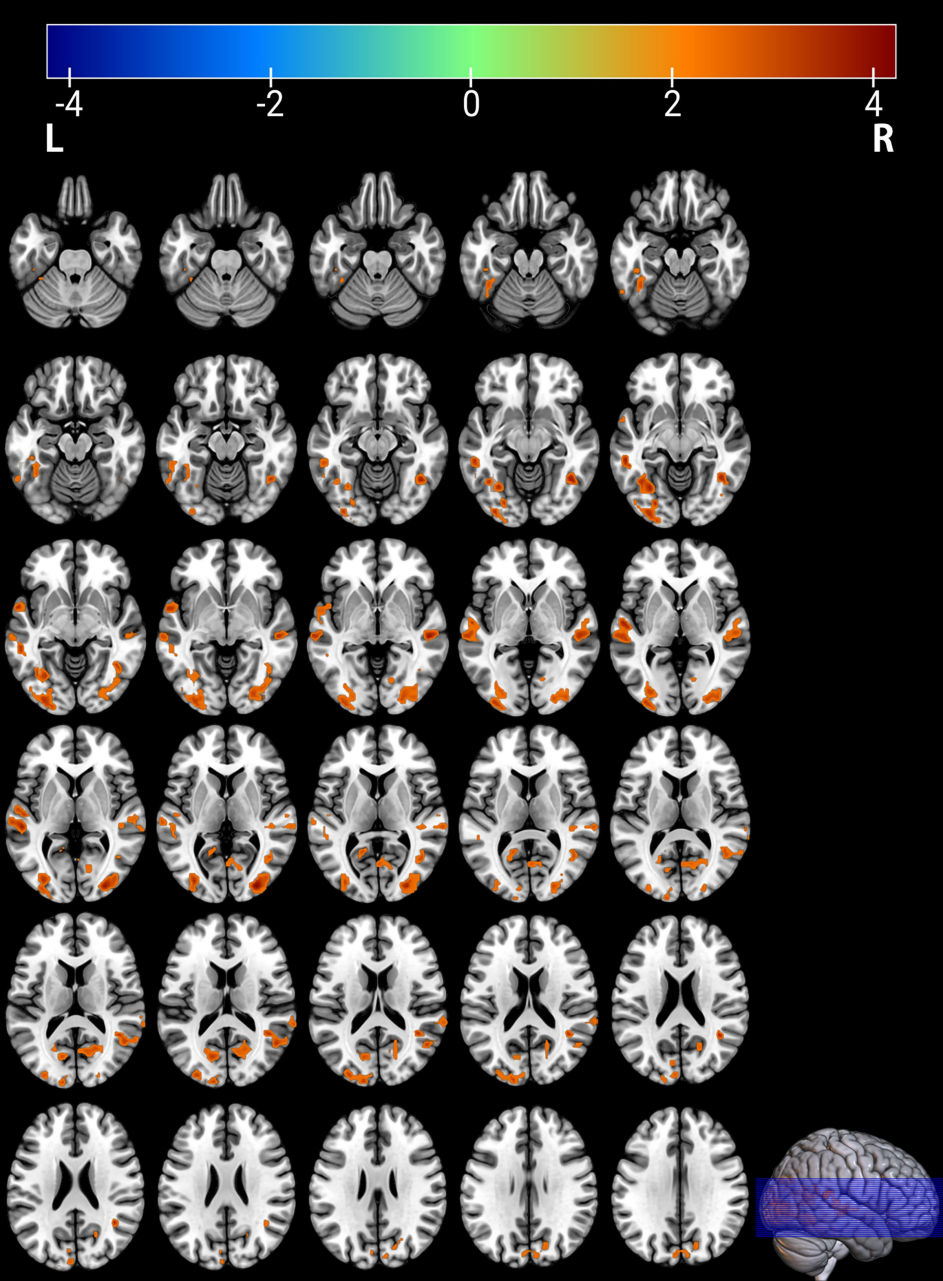
Brain regions with significantly increased ALFF in the PT group compared to the HC group. The results are displayed on 2D axial slices for detailed anatomical localization. Statistical significance was determined by a two-sample t-test, with voxel-wise FDR correction at *p* < 0.05. The color bar represents the T-value. Warm colors (red) indicate a significant increase in ALFF in the PT group. Significant clusters include the left fusiform gyrus (Fusiform_L), left middle temporal gyrus (Temporal_Mid_L), right middle occipital gyrus (Occipital_Mid_R), right middle temporal gyrus (Temporal_Mid_R), right calcarine cortex (Calcarine_R), right angular gyrus (Angular_R), and left calcarine cortex (Calcarine_L). R, right; L, left.

Comparison between the depressed PT group and the healthy HC group revealed differences in seven brain regions between the two groups (*p* < 0.05, FDR corrected, Table 2), with Fusiform_L, Temporal_Mid_L, Occipital_Mid_R, Temporal_Mid_R, Calcarine_R, Angular_R, and Calcarine_L increased (Figures 1, 2).

**Table 2:** ALFF two-sample *t*-test results report.

Peak point brain region	MNI peak coordinates			T value	Cluster size
	X	Y	Z		
Fusiform_L (aal)	−30	−66	−9	3.7348	338
Temporal_Mid_L (aal)	−54	−30	3	4.125	176
Occipital_Mid_R (aal)	30	−90	6	4.3296	195
Temporal_Mid_R (aal)	51	−27	−3	4.0037	114
Calcarine_R (aal)	21	−66	12	3.1043	93
Angular_R (aal)	39	−51	24	3.8749	81
Calcarine_L (aal)	−15	−72	15	3.415	91

### 3.3 Difference in fALFF between the two groups

**Figure 3:**
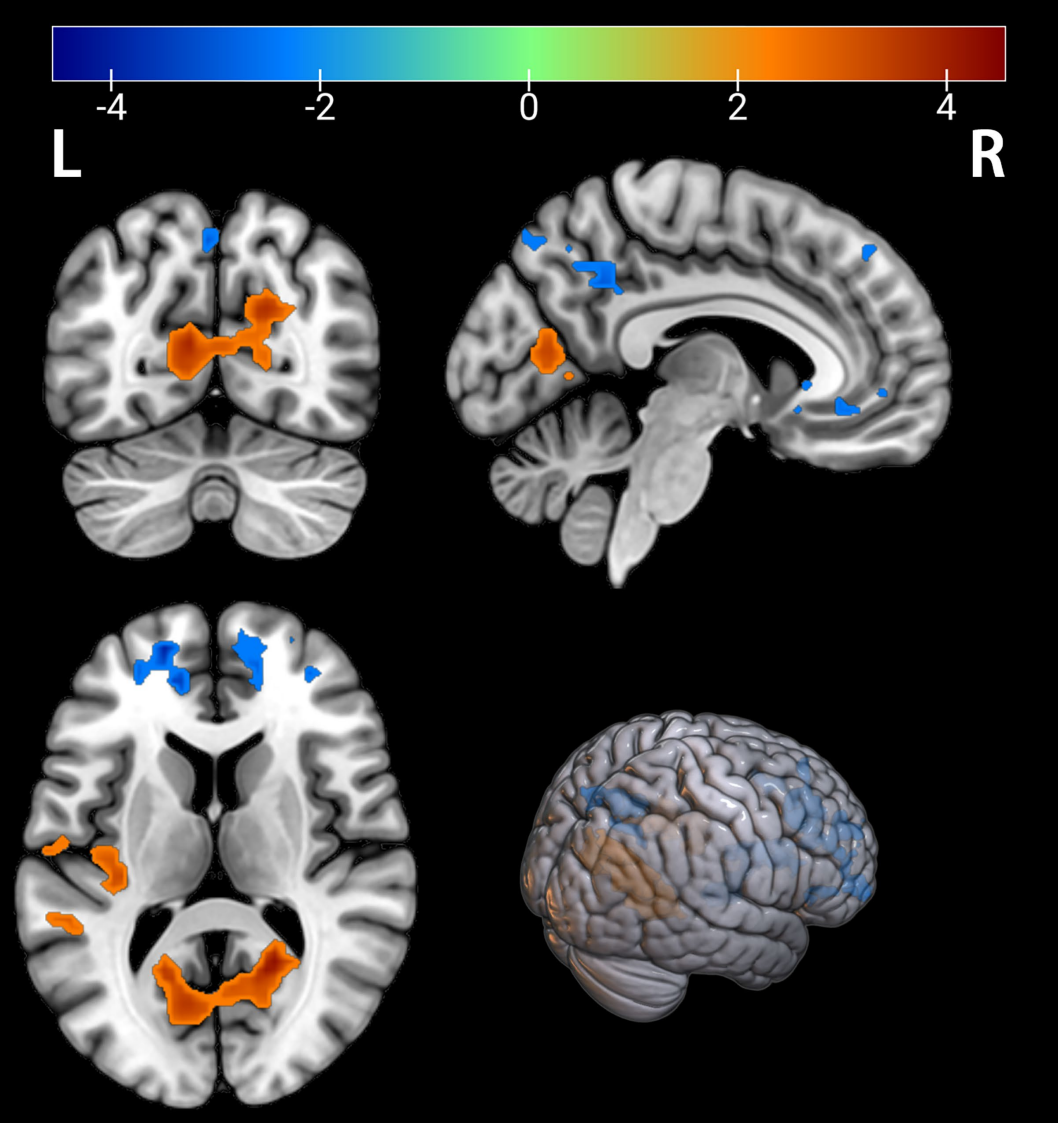
Brain regions with significant differences in fALFF between the PT and HC groups. The results are displayed on a 3D brain template. Statistical significance was determined by a two-sample t-test, with voxel-wise FDR correction at *p* < 0.05. The color bar represents the T-value. Warm colors (red) indicate a significant increase in fALFF in the PT group, and cool colors (blue) indicate a significant decrease. Significant clusters with increased fALFF include the right calcarine cortex (Calcarine_R) and left superior temporal gyrus (Temporal_Sup_L). Clusters with decreased fALFF include the left superior temporal pole (Temporal_Pole_Sup_L), right medial superior frontal gyrus (Frontal_Sup_Medial_R), left superior frontal gyrus (Frontal_Sup_L), and left precuneus (Precuneus_L). R, right; L, left.

**Figure 4:**
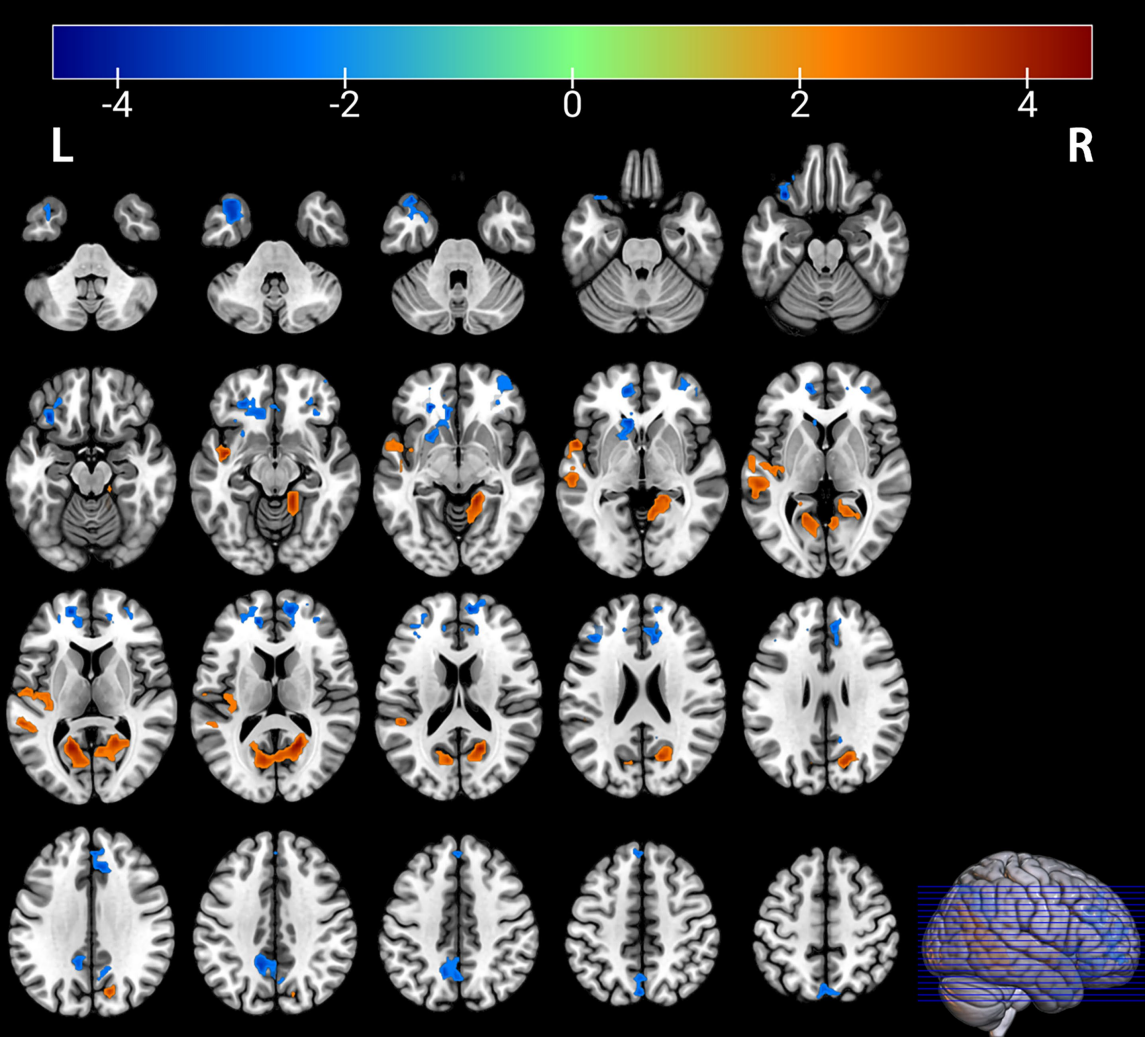
Brain regions with significant differences in fALFF between the PT and HC groups. The results are displayed on 2D axial slices for detailed anatomical localization. Statistical significance was determined by a two-sample t-test, with voxel-wise FDR correction at *p* < 0.05. The color bar represents the T-value. Warm colors (red) indicate a significant increase in fALFF in the PT group, and cool colors (blue) indicate a significant decrease. Significant clusters with increased fALFF include the right calcarine cortex (Calcarine_R) and left superior temporal gyrus (Temporal_Sup_L). Clusters with decreased fALFF include the left superior temporal pole (Temporal_Pole_Sup_L), right medial superior frontal gyrus (Frontal_Sup_Medial_R), left superior frontal gyrus (Frontal_Sup_L), and left precuneus (Precuneus_L). R, right; L, left.

Comparison of the depressed PT group with the healthy HC group revealed differences in seven brain regions between the two groups (*p* < 0.05, FDR corrected, Table 3), with an increase in the Calcarine_R, Temporal_Sup_L brain regions (Figures 3, 4); and a Temporal_Pole_Sup_L, Frontal_Sup_Medial_R, Frontal_Sup_L, Precuneus_L brain regions decreased (Figures 3, 4).

**Table 3:** fALFF two-sample *t*-test results report.

Peak point brain region	MNI peak coordinates			T value	Cluster size
	X	Y	Z		
Temporal_Pole_Sup_L (aal)	−33	24	−24	−4.4898	272
Calcarine_R (aal)	18	−57	15	4.6441	540
Temporal_Sup_L (aal)	−54	6	−3	4.1843	271
Frontal_Sup_Medial_R (aal)	9	57	18	−3.9658	245
Frontal_Sup_L (aal)	−18	57	9	−4.6183	117
Precuneus_L (aal)	−12	−51	36	−4.3801	165

### 3.4 ICA analysis of differences

**Figure 5:**
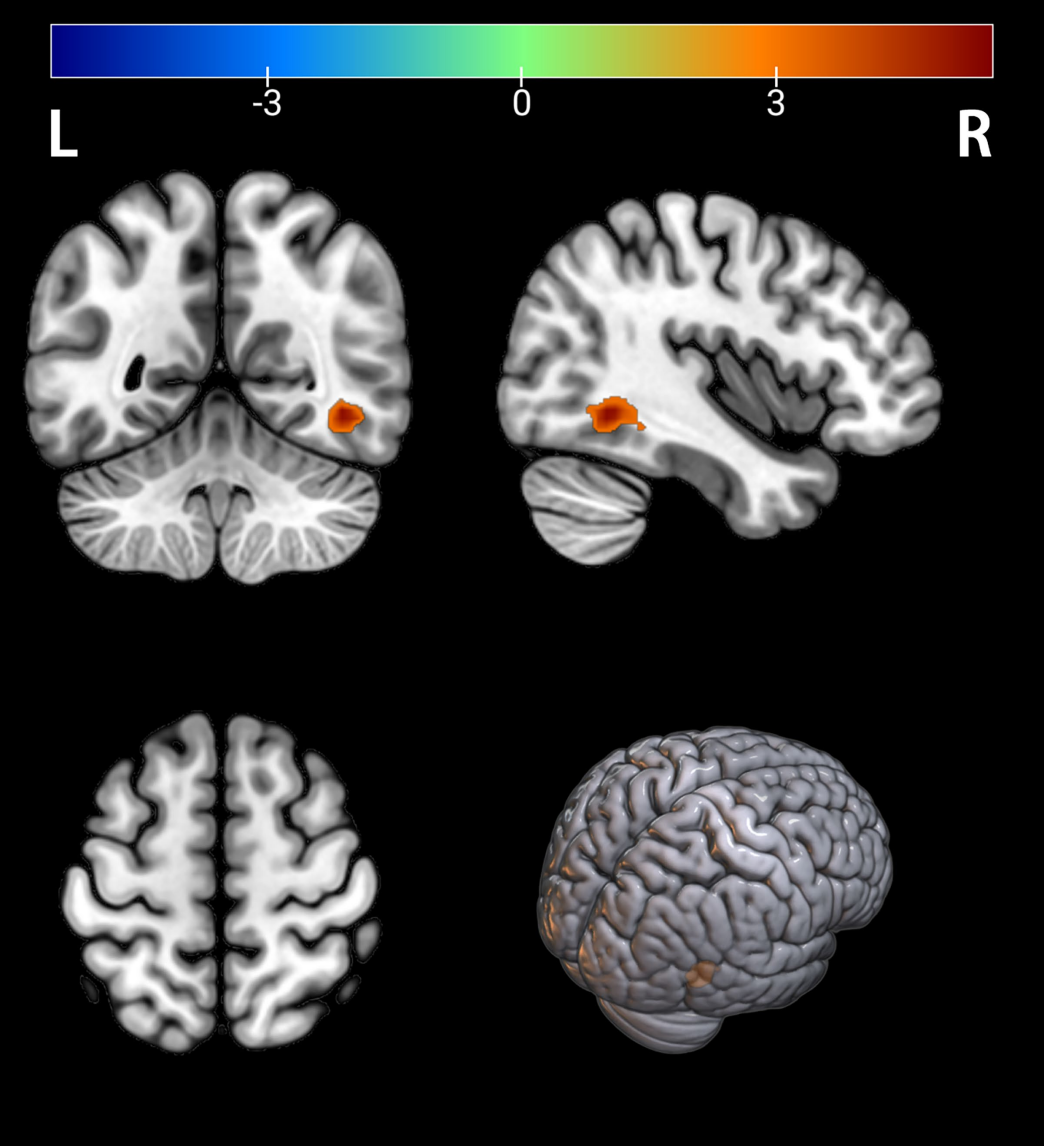
Brain regions with significant differences in functional connectivity within the VIN between the PT and HC groups. The results are displayed on a 3D brain template. Statistical significance was determined by a two-sample t-test (FWE-corrected at the cluster level with a voxel-wise initial threshold of *p* < 0.001). The color bar represents the T-value. Warm colors (red) indicate a significant increase in functional connectivity in the PT group. The peak of the significant cluster is located in the right inferior temporal gyrus (Temporal_Inf_R). R, right; L, left.

**Figure 6:**
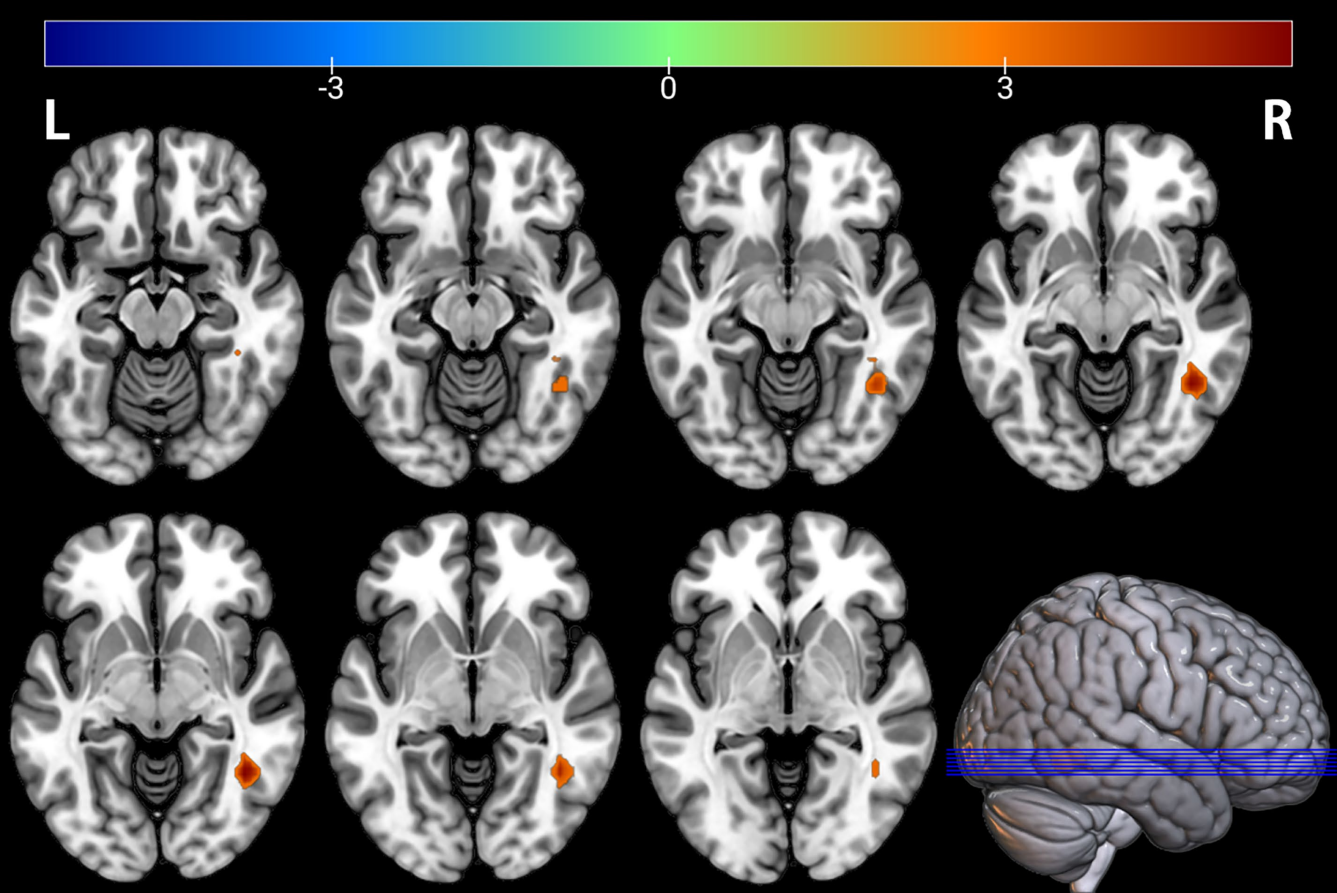
Brain regions with significant differences in functional connectivity within the visual network (VIN) between the PT and HC groups. The results are displayed on 2D axial slices for detailed anatomical localization. Statistical significance was determined by a two-sample t-test (FWE-corrected at the cluster level with a voxel-wise initial threshold of *p* < 0.001). The color bar represents the T-value. Warm colors (red) indicate a significant increase in functional connectivity in the PT group. The peak of the significant cluster is located in the right inferior temporal gyrus (Temporal_Inf_R). R, right; L, left.

There were differences in connectivity within the visual network (VIN) between the two groups, with the peak point of difference being in the Temporal_Inf_R brain region (Table 4), and elevated connectivity in the depressed group in the Temporal_Inf_R brain region (FWE-corrected, voxel *p* < 0.001, cluster *p* < 0.05, Figures 5, 6).

**Table 4:** Reported results of two-sample *t*-tests within the VIN_15 network.

Peak point brain region	MNI peak coordinates			T value	Cluster size
	X	Y	Z		
Temporal_Inf_R (aal)	42	−51	−6	5.5899	44

## 4 Discussion

In this study, we exploratorily analyzed the differences in fALFF and ALFF values between adolescents with first-episode depression and normal controls by means of resting-state functional magnetic resonance imaging (rs-fMRI), focusing on the left temporal pole supratemporal gyrus (Temporal_Pole_Sup_L), bilateral talar fissure (Calcarine), left temporal supramedial gyrus (Temporal_ Sup_L), bilateral middle temporal gyrus (Temporal_Mid), right medial supramedial frontal gyrus (Frontal_Sup_Medial_R), left supramedial frontal gyrus (Frontal_Sup_L), and left precuneus_L), left fusiform gyrus (Fusiform_L), right occipital gyrus (Occipital The neural activity characteristics of the brain regions of the left precuneus (Precuneus_L), left fusiform gyrus (Fusiform_L), right middle occipital gyrus (Occipital_R) and right angular gyrus (Angular_R). The results showed that there were significant abnormalities in the intensity of spontaneous neural activity (ALFF) and standardized low frequency amplitude (fALFF) in the above brain regions, suggesting that the pathological mechanism of adolescent depression may involve the synergistic dysregulation of multiple functional networks.

The temporal pole, as a key node for social cognition and emotional integration, plays an important role in the development of depression (Sang et al., 2025). In addition, the superior temporal gyrus is involved in auditory information processing and language-related emotion regulation, and changes in its function may affect the efficiency of patients’ processing of negative emotional stimuli (Wu et al., 2024). Previous studies have also shown that the left temporal lobe exhibits higher discriminative ability in detecting MDD, highlighting its important role in the neurobiology of depression (Sang et al., 2025). In the present study, we found that the abnormal temporal lobe changes in adolescent depressed patients were all concentrated in the left brain region, and the patients showed significant abnormalities in the fALFF values of the left temporal pole temporal supramarginal gyrus (Temporal_Pole_Sup_L) and temporal gyrus superiorly (Temporal_Sup_L) as well as the ALFF values of the left temporal middle gyrus (Temporal_Mid_L), and in our speculation that their activity The abnormalities may be related to emotional blunting and social withdrawal in adolescent patients, further demonstrating the importance and diagnostic value of the left temporal lobe in adolescent depression.

Notably, the right talar fissure (Calcarine_R) is an important region for visual and auditory processing, participating in multiple independent networks and involved in multisensory processing in vision, hearing, language and emotion (Wei et al., 2019). In the present study, elevated fALFF values were found in the right talar fissure (Calcarine_R), a phenomenon that may be associated with excessive attention to negative visual information in depressed patients. Yao et al. similarly found that the talar fissure was markedly dysregulated in depressed patients, which was improved by the administration of antidepressant medication, suggesting a direct role in the relationship between this region and depression (Yao et al., 2022). Previous studies have suggested that enhanced functional connectivity between emotion-related brain regions and the visual cortex may exacerbate sensitivity to negative stimuli, a finding that provides support for the present findings (Zhao et al., 2022).

ALFF values were significantly lower in the right medial supra-frontal (Frontal_Sup_Medial_R) and left supra-frontal gyrus (Frontal_Sup_L). The medial frontal is a core region of the default mode network (DMN), and its under-inhibited activity may lead to excessive self-referential thinking, which is closely related to the tendency of rumination in depressed patients (Carhart-Harris et al., 2017; Sezer et al., 2022). In contrast, the reduced ALFF values in the supra-frontal gyrus may reflect impaired executive functioning in adolescent patients, especially reduced emotion regulation and cognitive flexibility. This result is consistent with the findings of Gong et al. (2020) in adult depression, suggesting that abnormal prefrontal function may be a common feature of depression across age groups. In addition, high-frequency repetitive transcranial magnetic stimulation of frontal subregions significantly reduced anxiety scores in patients with anxiety disorders by Herrmann et al. (2017), suggesting that frontal subregions may play a key role in anxiety-related depression.

The present study found elevated fALFF values in the left precuneus (Precuneus_L), a finding similar to that of Liu et al. (2025) in patients with first-episode major depressive disorder, and this elevated neuronal activity may suggest hyperarousal of neural activity in this region in adolescent depression. The precuneus, as an important component of the DMN, is involved in the integration of self-awareness and situational memory. Its enhanced activity may reflect adolescent patients’ fixation on negative self-perceptions, such as overly focusing on their own failure experiences or holding pessimistic expectations about the future. Similarly, Wang et al. (2025) found that enhanced precuneus functional connectivity was significantly associated with negative self-appraisal in depressed patients. Crane et al. reported increased precuneus activation in anxious depression, which may be related to its involvement in self-consciousness, and all these studies further validate the important influence of the precuneus in depression (Crane et al., 2016).

A large number of previous rs-fMRI studies have found the presence of changes in the left fusiform gyrus (Fusiform_L) in patients with depression (Liu et al., 2023). Gong et al. demonstrated significant alterations in overall functional connectivity density in the bilateral fusiform region in patients with MDD (Gong et al., 2017). Van Geest et al. (2019) likewise suggested the presence of abnormalities in the fusiform gyrus in patients, which is similar to the findings of the present study, suggesting changes in neural activity in this brain region, which may underlie depression. And Li et al.’s ALFF study of the right middle occipital gyrus in treated MDD patients suggested that abnormal activity in the middle occipital gyrus may be associated with depression and suicidal symptoms in adolescents, which is similar to the activity in the right middle occipital gyrus (Occipital_Mid_R) in adolescent manoeuvre depression patients in the present study, suggesting that the right middle occipital gyrus may be a characteristic manifestation of depression in adolescents (Li et al., 2022).

Several limitations of this study should be acknowledged. First, the sample size, while reasonable for an initial exploratory study, remains modest for whole-brain voxel-wise analyses and may limit the generalizability and statistical power of our findings. Future studies with larger, multi-center samples are needed for replication and validation. Second, the cross-sectional design limits the ability to draw causal inferences regarding the observed neural alterations and their potential role in the development of depression. Third, while several key covariates were controlled for, other potentially influential factors—such as sleep quality, attentional state during neuroimaging, and intelligence quotient (IQ)—were not assessed and may represent unmeasured confounders. Fourth, the selection of functional networks based on independent component analysis (ICA) results, although guided by established templates, inherently involves a degree of methodological subjectivity. Finally, the non-normal distribution of clinical scale scores among healthy controls highlights the necessity of employing appropriate non-parametric or distribution-robust statistical approaches. Despite these limitations, the current study offers a comprehensive multi-parametric characterization of brain function in adolescents with first-episode depression (FED), providing a foundation for future longitudinal and interventional research.

## 5 Conclusion

In summary, the neurological abnormalities in adolescents with first-episode depression involve the temporal lobe emotion processing network, the prefrontal executive control system, and the default mode network. Abnormalities of low-frequency activity in the temporal pole and superior frontal gyrus may exacerbate impaired emotion regulation, whereas hyperactivation of the precuneus with the visual cortex may reinforce negative self-perception. Our exploratory findings suggest that the right middle occipital gyrus may represent a potential characteristic neurobiological feature of adolescent depression worthy of further investigation. These findings provide new perspectives for understanding the early neural mechanisms of adolescent depression and suggest that non-invasive neuromodulation techniques (e.g., transcranial magnetic stimulation) targeting specific brain regions may have therapeutic potential.

## Data Availability

The raw data supporting the conclusions of this article will be made available by the authors, without undue reservation.

## Footnotes

^1^ http://www.restfmri.net

^2^ http://mialab.mrn.org/software/gift/

## References

[ref1] Carhart-Harris R. L. Roseman L. Bolstridge M. Demetriou L. Pannekoek J. N. Wall M. B. . (2017). Psilocybin for treatment-resistant depression: fmri-measured brain mechanisms. Sci. Rep. 7:13187. doi: 10.1038/s41598-017-13282-7, PMID: 29030624 PMC5640601

[ref2] Chen H. Ding C. Ren J. (2025). The burden and trends of depressive disorders in adolescent and young adults aged 15-29 in China, 1990-2021 and its prediction to 2030: findings from the global burden of disease study 2021. J. Affect. Disord. 379, 594–604. doi: 10.1016/j.jad.2025.03.054, PMID: 40086484

[ref3] Crane N. A. Jenkins L. M. Dion C. Meyers K. K. Weldon A. L. Gabriel L. B. . (2016). Comorbid anxiety increases cognitive control activation in major depressive disorder. Depress. Anxiety 33, 967–977. doi: 10.1002/da.22541, PMID: 27454009 PMC5050098

[ref4] GBD 2021 Diseases and Injuries Collaborators (2024). Global incidence, prevalence, years lived with disability (Ylds), disability-adjusted life-years (Dalys), and healthy life expectancy (Hale) for 371 diseases and injuries in 204 countries and territories and 811 subnational locations, 1990-2021: a systematic analysis for the global burden of disease study 2021. Lancet 403, 2133–2161. doi: 10.1016/S0140-6736(24)00757-8, PMID: 38642570 PMC11122111

[ref5] Gong L. He C. Yin Y. Ye Q. Bai F. Yuan Y. . (2017). Nonlinear modulation of interacting between Comt and depression on brain function. Eur. Psychiatry 45, 6–13. doi: 10.1016/j.eurpsy.2017.05.024, PMID: 28728097

[ref6] Gong J. Wang J. Qiu S. Chen P. Luo Z. Wang J. . (2020). Common and distinct patterns of intrinsic brain activity alterations in major depression and bipolar disorder: voxel-based meta-analysis. Transl. Psychiatry 10:353. doi: 10.1038/s41398-020-01036-5, PMID: 33077728 PMC7573621

[ref7] Gore F. M. Bloem P. J. Patton G. C. Ferguson J. Joseph V. Coffey C. . (2011). Global burden of disease in young people aged 10-24 years: a systematic analysis. Lancet 377, 2093–2102. doi: 10.1016/S0140-6736(11)60512-6, PMID: 21652063

[ref8] Guo Y. Wei D. (2025). The functional connectivity between dorsal-medial prefrontal cortex and middle cingulate cortex links trait rumination and depressive symptoms. PLoS One 20:e0328895. doi: 10.1371/journal.pone.0328895, PMID: 40758674 PMC12321059

[ref9] Herrmann M. J. Katzorke A. Busch Y. Gromer D. Polak T. Pauli P. . (2017). Medial prefrontal cortex stimulation accelerates therapy response of exposure therapy in acrophobia. Brain Stimul. 10, 291–297. doi: 10.1016/j.brs.2016.11.007, PMID: 27931887

[ref10] Kaiser R. H. Andrews-Hanna J. R. Wager T. D. Pizzagalli D. A. (2015). Large-scale network dysfunction in major depressive disorder: a Meta-analysis of resting-state functional connectivity. JAMA Psychiatry 72, 603–611. doi: 10.1001/jamapsychiatry.2015.0071, PMID: 25785575 PMC4456260

[ref11] Li X. Chen X. Zhou Y. Dai L. Cui L. B. Yu R. . (2022). Altered regional homogeneity and amplitude of low-frequency fluctuations induced by electroconvulsive therapy for adolescents with depression and suicidal ideation. Brain Sci. 12:1121. doi: 10.3390/brainsci12091121, PMID: 36138857 PMC9496677

[ref12] Li R. Zhang Y. Zhang H. Wang C. Duan H. Sun S. . (2025). Crmp2 in the hippocampus alleviates chronic stress-induced depressive-like behaviours in mice by affecting synaptic function. Behav. Brain Res. 484:115495. doi: 10.1016/j.bbr.2025.115495, PMID: 40020760

[ref13] Liang N. Xue Z. Xu J. Sun Y. Li H. Lu J. (2025). Abnormal resting-state functional connectivity in adolescent depressive episodes. Psychiatry Res. Neuroimaging 348:111961. doi: 10.1016/j.pscychresns.2025.111961, PMID: 39983531

[ref14] Liu M. Huang Y. Li X. Liu Y. Yu R. Long Y. . (2022). Aberrant frontolimbic circuit in female depressed adolescents with and without suicidal attempts: a resting-state functional magnetic resonance imaging study. Front. Psychol. 13:1007144. doi: 10.3389/fpsyt.2022.1007144, PMID: 36386991 PMC9641155

[ref15] Liu C. Li H. Feng S. Zhang Z. Huang M. Lin S. . (2025). Alterations in structural and functional magnetic resonance imaging associated with cognitive function in patients with treatment-naïve first-episode major depressive disorder. Prog. Neuro-Psychopharmacol. Biol. Psychiatry 139:111367. doi: 10.1016/j.pnpbp.2025.111367, PMID: 40246055

[ref16] Liu J. Shu Y. Wu G. Hu L. Cui H. (2023). A neuroimaging study of brain activity alterations in treatment-resistant depression after a dual target accelerated transcranial magnetic stimulation. Front. Psychol. 14:1321660. doi: 10.3389/fpsyt.2023.1321660, PMID: 38288056 PMC10822961

[ref17] Macdonald S. E. Becker C. R. Macnamara A. (2025). Amygdala-insula response to neutral stimuli and the prospective prediction of anxiety sensitivity. Prog. Neuro-Psychopharmacol. Biol. Psychiatry 139:111384. doi: 10.1016/j.pnpbp.2025.111384, PMID: 40300661 PMC12810358

[ref18] Sang Q. Chen C. Shao Z. (2025). Decoding depression from different brain regions using hybrid machine learning methods. Bioengineering (Basel) 12:449. doi: 10.3390/bioengineering12050449, PMID: 40428068 PMC12109470

[ref19] Sezer I. Pizzagalli D. A. Sacchet M. D. (2022). Resting-state fmri functional connectivity and mindfulness in clinical and non-clinical contexts: a review and synthesis. Neurosci. Biobehav. Rev. 135:104583. doi: 10.1016/j.neubiorev.2022.104583, PMID: 35202647 PMC9083081

[ref20] Van Geest Q. Boeschoten R. E. Keijzer M. J. Steenwijk M. D. Pouwels P. J. Twisk J. W. . (2019). Fronto-limbic disconnection in patients with multiple sclerosis and depression. Mult. Scler. 25, 715–726. doi: 10.1177/1352458518767051, PMID: 29587565 PMC6439942

[ref21] Wang S. Chen C. Wang J. Wang R. the REST‐meta‐MDD Consortium Mao F. . (2025). Transcriptional patterns of functional connectivity associated with somatic symptoms in major depressive disorder. Am. J. Med. Genet. B Neuropsychiatr. Genet.:e33041. doi: 10.1002/ajmg.b.33041, PMID: 40546215

[ref22] Wang H. Y. You H. L. Song C. L. Zhou L. Wang S. Y. Li X. L. . (2024). Shared and distinct prefrontal cortex alterations of implicit emotion regulation in depression and anxiety: an fnirs investigation. J. Affect. Disord. 354, 126–135. doi: 10.1016/j.jad.2024.03.032, PMID: 38479517

[ref23] Wei H. L. Zhou X. Chen Y. C. Yu Y. S. Guo X. Zhou G. P. . (2019). Impaired intrinsic functional connectivity between the thalamus and visual cortex in migraine without aura. J. Headache Pain 20:116. doi: 10.1186/s10194-019-1065-1, PMID: 31856703 PMC6924083

[ref24] Wu B. Zhang X. Xie H. Wang X. Gong Q. Jia Z. (2024). Disrupted structural brain networks and structural-functional decoupling in first-episode drug-naïve adolescent major depressive disorder. J. Adolesc. Health 74, 941–949. doi: 10.1016/j.jadohealth.2024.01.015, PMID: 38416102

[ref25] Yao G. Zhang X. Li J. Liu S. Li X. Liu P. . (2022). Improving depressive symptoms of post-stroke depression using the Shugan Jieyu capsule: a resting-state functional magnetic resonance imaging study. Front. Neurol. 13:860290. doi: 10.3389/fneur.2022.860290, PMID: 35493835 PMC9047823

[ref26] Zhang H. Zhang L. Lu J. Yue J. Yuan Z. Hu J. . (2025). Multiparameter resting-state functional magnetic resonance imaging as an indicator of neuropsychological changes in Binswanger's disease with mild cognitive impairment. Front. Aging Neurosci. 17:1522591. doi: 10.3389/fnagi.2025.1522591, PMID: 39995946 PMC11847846

[ref27] Zhang W. Zhang C. Zhao J. Cui J. Bai J. Deng X. . (2025). Microstructure abnormalities of diffusion tensor imaging measures in first-episode, treatment-naïve adolescents with major depressive disorder: an integrated Afq and Tbss study. Brain Behav. 15:e70416. doi: 10.1002/brb3.70416, PMID: 40079635 PMC11905106

[ref28] Zhao N. Yue J. Feng Z. J. Qiao Y. Ge Q. Yuan L. X. . (2022). The location reliability of the resting-state fmri fc of emotional regions towards rtms therapy. Neuroinformatics 20, 1055–1064. doi: 10.1007/s12021-022-09585-4, PMID: 35608748

